# Pathogenesis of *Isospora amphiboluri* in Bearded Dragons (*Pogona vitticeps*)

**DOI:** 10.3390/ani11020438

**Published:** 2021-02-08

**Authors:** Michael Walden, Mark A. Mitchell

**Affiliations:** School of Veterinary Medicine, Louisiana State University, Skip Bertman Drive, Baton Rouge, LA 70803, USA; corsnakevet@yahoo.com

**Keywords:** bearded dragon, coccidia, *Isospora amphiboluri*, pathogenesis, *Pogona vitticeps*

## Abstract

**Simple Summary:**

Coccidia are common parasites of captive animals. While there have been a number of studies evaluating the life cycles of these parasites in domestic pets and livestock, there has been limited research assessing the impact of these parasites on reptiles. Bearded dragons are a common pet lizard and are known to be infected by their own species of coccidia, *Isospora amphiboluri*. To determine the best practices for controlling this parasite in captive bearded dragons, it is important that we learn about what the parasite does once it infects the bearded dragon. This study found that *Isospora amphiboluri* infects the small and large intestines of bearded dragons. In addition, the time (pre-patent period) from exposure to shedding the parasite in feces is 15–22 days. This information is important for developing treatment and management protocols for captive bearded dragons to reduce their exposure to this parasite.

**Abstract:**

*Isospora amphiboluri* is a common coccidian found in captive bearded dragons (*Pogona vitticeps*). To minimize the impact of this parasite, it is important to characterize its pathogenesis so that we can develop appropriate methods for diagnosis and treatment. Forty-five juvenile bearded dragons were used for this two-part study. In the first part, ten bearded dragons were infected with 20,000 oocysts per os, while a control group of five animals received only water. Feces were collected over 45 days and screened for oocysts. In the second part, thirty bearded dragons were used to characterize the pathogenesis of *I. amphiboluri*. Twenty-five bearded dragons were infected as described previously, while five animals served as controls. Five infected bearded dragons and one control were humanely euthanized on days 4, 8, 12, 16, and 20 post-infection for complete necropsies. The pre-patent period for *I. amphiboluri* was found to be 18.6 ± 1.9 days (range 15–22 days). Histopathology confirmed that *I. amphiboluri* follows a homoxenous life cycle. Infections begin in the duodenum and progress to the colon over time. The findings of this study can be used to develop better quarantine and treatment protocols for captive bearded dragons.

## 1. Introduction

*Isospora* is an important parasite of animals, and while there have been a number of studies characterizing the life cycle of this coccidian in domestic animals [1,2,3], it has been poorly studied in reptiles. Studies in reptiles have generally been limited to basic investigation into the life stages of *Isospora* spp. [4,5,6,7,8,9,10,11,12,13,14,15,16,17,18,19,20,21,22,23], with most being focused on the description of oocysts from new species or the fine structure of various life stages without delving into the more medically relevant discussion of prepatent period, tissue or organ tropism, and pathologic changes associated with infection. By developing an understanding of these aspects of the life cycle of *Isospora* spp. in reptiles, veterinarians and herpetoculturists can develop more appropriate methods for managing these organisms in captive reptile populations.

In mammals, the genus *Isospora* was originally thought to have a homoxenous (monoxenous) life cycle, being confined to one host and the intestine of the host animals [3]. However, Frenkel and Dubey [24] demonstrated that rodents could act as intermediate hosts by ingesting *Isospora* oocysts from infected cats. Based on these findings, Dubey [25] proposed a new generic name, *Levineia*, for organisms that can use intermediate hosts and develop outside of the intestinal tract. However, this new classification has not been widely adopted, and the genus *Isospora* remains a complicated one with at least three distinct types of life cycles. One type involves both the use of an intermediate host and extra-intestinal stages. This life cycle is illustrated by the mammalian *Isospora felis*, which may use rodents as intermediate hosts and can form extra-intestinal stages in intermediate and definitive hosts [3,25,26]. Another life cycle is illustrated by *Isospora serini* in the canary, which has life stages outside of the intestine but no known intermediate host [3,27]. The final described life cycle is gut-limited, with all life stages occurring in the intestines. This cycle appears to be common in reptiles. In recent years, several new species of *Isospora* have been described in lizards; these species possess the ability to produce a gut-limited homoxenous life cycle, and they include *Isospora ameivae* from the teiid *Ameiva ameiva* [13]; *Isospora capanemaensis* from the amphisbaenian *Amphisbaena alba* [19]; *Isospora carliae* from the skink *Carlia rhomboidalis* [23]; *Isospora cryptoblephari* and *Isospora delmae* from skinks [28]; and *Isospora gehyrae*, *Isospora cytoheteronotis*, *Isospora nucleoheteronotis*, and *Isospora oedurae* from Australian geckos [18].

*Isospora amphiboluri* is an important and widespread parasite of commercially bred central bearded dragons (*Pogona vitticeps*). This coccidian has been associated with up to 15% mortality in young lizards [29]. *I. amphiboluri* was first described in Eastern bearded dragons (*Pogona barbata*) by Cannon [5]. Since then, it was described again by McAllister et al. [9] in *P. vitticeps*, as well as a co-pathogen in *P. vitticeps* with adenovirus and dependovirus [29]. However, in the 1995 re-description of the species, it was unclear if tissues other than intestines were examined, and the study by Kim et al. [29] focused more on the viral infection and did not perform a thorough examination of the coccidian life cycle. To date, no studies have described the pathologic changes associated with *I. amphiboluri* as a single infecting organism, addressed the prepatent period, or described the localization of the parasite in host tissues. The purpose of this study was to examine these clinically relevant questions. The specific hypotheses being tested in this study were: (1) *I. amphiboluri* infections follow a gut-limited homoxenous life cycle, (2) the pre-patent period of this parasite will be approximately 18 days, (3) there will be infection site specificity within the intestine, (4) the distribution of life stages will become greater over the course of infection, and (5) the pathologic changes will become greater over the course of infection.

## 2. Materials and Methods

### 2.1. Animals

Forty-five juvenile (4–6 months of age) central bearded dragons were used for this study. The animals were recruited as being negative for coccidia following treatment with oral ponazuril (Bayer Animal Health Division, Shawnee Mission, KS, USA) at 30 mg/kg (once daily for 7 days) and being monitored for 45 days with daily fecal floatation examinations using modified Sheather’s solution (sucrose, water, and 6 mL of phenol with a solution-specific gravity of 1.26). All animals were negative for coccidia oocysts for the full 45 days. The animals were individually housed at 30 °C in 32 × 18 × 12 cm^3^ plastic boxes with paper substrate. Fecal material was removed daily, and the cages were completely replaced every two weeks. All animals were fed the same diet, which consisted of house crickets (*Acheta domesticus*), mealworms (*Tenebrio molitor*), and washed shredded collard (*Brassica oleracea*), mustard (*Brassica juncea*), and turnip (*Brassica rapa*) greens. The invertebrates and greens were acquired from Fluker Farms (Port Allen, LA, USA) and a local grocery store, respectively.

### 2.2. Collecting and Processing Oocysts

**Figure 1:**
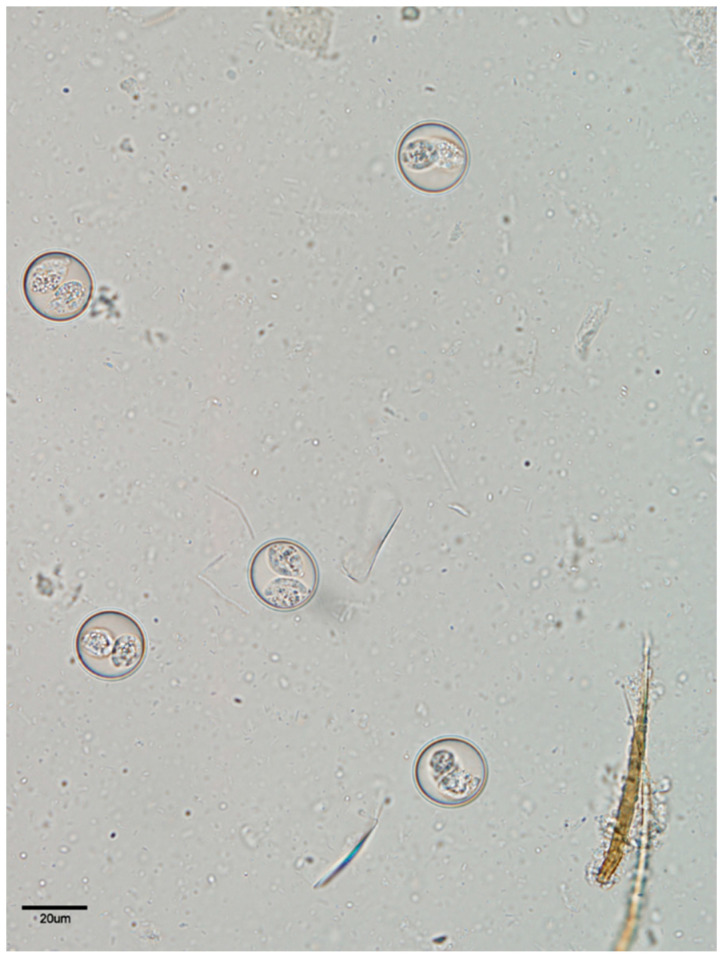
Oocysts of *Isospora amphiboluri* from modified Sheather’s solution fecal floatation. Magnification at 600×.

Feces from *I. amphiboluri* infected dragons were collected and the number of oocysts recorded. Oocysts were spherical to slightly subspherical, smooth, and had bilayered walls. The oocysts were 22.7–25.4 µm in diameter, and the oocyst wall measured 1.1–1.4 µm in thickness for a shape index (length/width) of 1.0–1.1. The micropyle, oocyst residuum, and polar granules were absent. Approximately 85% of the oocysts were sporulated upon the initial examination of the fecal samples. The sporulated oocysts contained two sporocysts measuring 11–17 µm in diameter. The sporocysts were single layered with a smooth wall, substiedal body, and a prominent Stieda body (Figure 1). After confirming the presence of the oocysts, the slides were washed with tap water and the samples centrifuged at 8000× *g* for 10 min to collect the oocysts. Multiple samples were pooled to create infection doses of 20,000 oocysts, with the variation among the doses not exceeding ±700 oocysts. The number of oocysts used as the infection dose represented a high dose of oocysts collected from the feces of infected bearded dragons in the authors’ laboratory. The samples were washed and centrifuged with tap water five more times, and the samples were left to sporulate for 48 h at room temperature. Five additional samples were floated and examined to ensure that sporulation had taken place. After 48 h of incubation at room temperature, all oocysts were found to have sporulated.

### 2.3. Prepatent Period Model

Fifteen bearded dragons were used to determine the prepatent period of *I. amphiboluri*. A random number generator (random.org) was used to divide the animals into two groups: infected (*n* = 10) and control (*n* = 5). Ten bearded dragons were each orally dosed with 20,000 ± 700 sporulated *I. amphiboluri* oocysts suspended in 0.5 mL of tap water, while the five control animals were given a placebo of 0.5 mL of tap water. Feces were collected once daily and screened for oocysts using modified Sheather’s solution fecal floatation. Fecal floatations were performed for 45 days or until all animals shed oocysts.

### 2.4. Pathogenesis Model

Thirty juvenile bearded dragons were used for the pathogenesis study. The animals were randomly divided into six groups of five animals using a random number generator (random.org). Group 6 served as the control group and received an oral placebo of 0.5 mL of tap water, while the remaining five groups received an oral dose of 20,000 ± 700 sporulated *I. amphiboluri* oocysts in 0.5 mL of tap water. Feces were collected daily from each animal. To characterize site localization and the pathogenesis of the organisms over time, the animals were euthanized at different intervals: Group 1 was euthanized 4 days post infection, Group 2 was euthanized 8 days post infection, Group 3 was euthanized 12 days post infection, Group 4 was euthanized 16 days post infection, and Group 5 was euthanized 20 days post infection. Times were selected based on the prepatent period results. One control animal was euthanized at the same time that each group was euthanized. The lizards were euthanized using a two-stage process. First, the animals received a dose of 50 mg/kg of ketamine (Ketaset, 100 mg/mL, Ft. Dodge Co., Ft. Dodge, IA, USA) intramuscularly. Once the animals had lost their righting reflex, 39 mg of pentobarbital (Beuthanasia, 390 mg/mL, Schering-Plough Animal Health Corp., Union, NJ, USA) were injected intracardiac to complete the euthanasia. A Doppler probe was used to confirm asystole. A complete necropsy was performed on each animal, and tissues were collected for histopathology.

Tissues were fixed in 10% buffered neutral formalin for at least 72 h before processing. Tissues were embedded in paraffin and four-µm-thick sections were cut for histologic examination. Tissues were stained with hematoxylin and eosin. The liver, heart, lungs, kidney, spleen, stomach, small intestine, colon, and skeletal muscle were examined histologically, and the life stages, location of life stages, and pathological changes were characterized and described. Each section of intestine was examined separately.

### 2.5. Statistical Analysis

The presence of life stages in sections of the gut were measured as dichotomous data (presence/absence). The 95% binomial confidence intervals (CI) were calculated for appropriate proportions. The distributions of continuous data (time for oocyst shedding) were evaluated using the Shapiro–Wilk test, skewness, kurtosis, and q–q plots. For purposes of analysis, the gastrointestinal tract was divided into four sites: duodenum, jejunum, ileum, and colon. A 4 × 2 chi square test was used to determine if there were any differences in tissue tropism for the parasite. The dependent variable was the presence/absence of parasites, and the independent variable was site (duodenum, jejunum, ileum, and colon). A 4 x 2 chi-square test was also used to determine if there were significant differences in the presence/absence of life stages at each site based on time. If differences were found, a Fisher’s exact test was used to characterize the differences in time. Statistical analyses were performed using SPSS 22 (SPSS Inc., IBM statistics, Armonk, NY, USA). A *p* < 0.05 value was used to determine statistical significance.

## 3. Results

### 3.1. Prepatent Period

**Figure 2:**
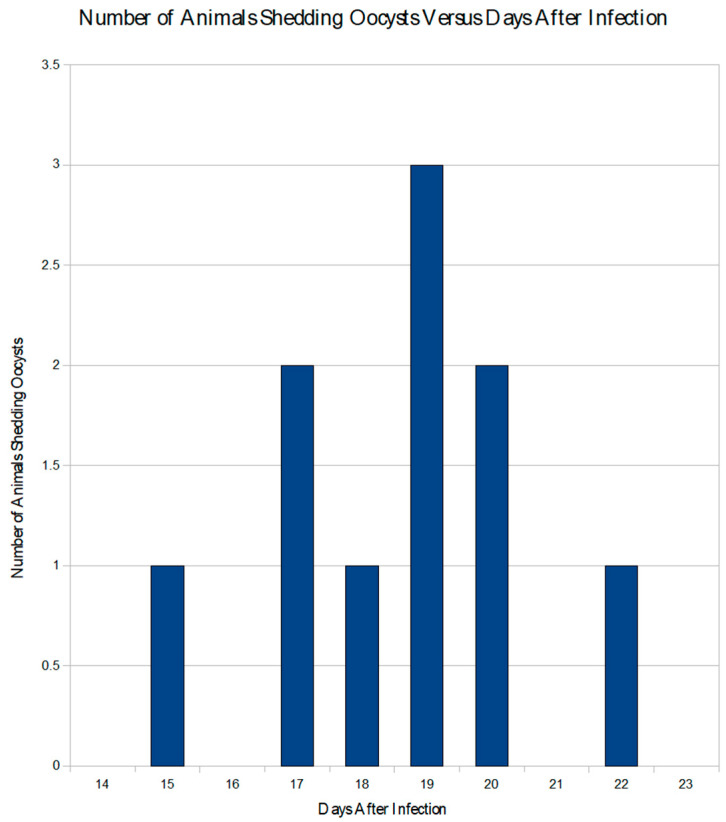
Graph showing the number of infected animals shedding oocysts in the prepatent period trial and the days oocyst shedding was detected.

In the prepatent period study, all of the animals (*n* = 10) infected with sporulated *I. amphiboluri* were found to shed oocysts (10/10), while all of the control animals (*n* = 5) remained negative for 45 days. The average prepatent period was 18.6 ± 1.9 days (range of 15–22 days) (Figure 2).

### 3.2. Pathogenesis of Isospora Amphiboluri

Animals in Groups 1–5 had detectable oocysts in their feces for the first two-to-three days post infection. There were no oocysts observed in the fecal samples of any group at three days post infection. Control group animals (*n* = 5) had no observed life stages in any of the examined tissues at any time point. There were no gross changes to the gastrointestinal tract of any bearded dragon at the time of necropsy.

**Figure 3:**
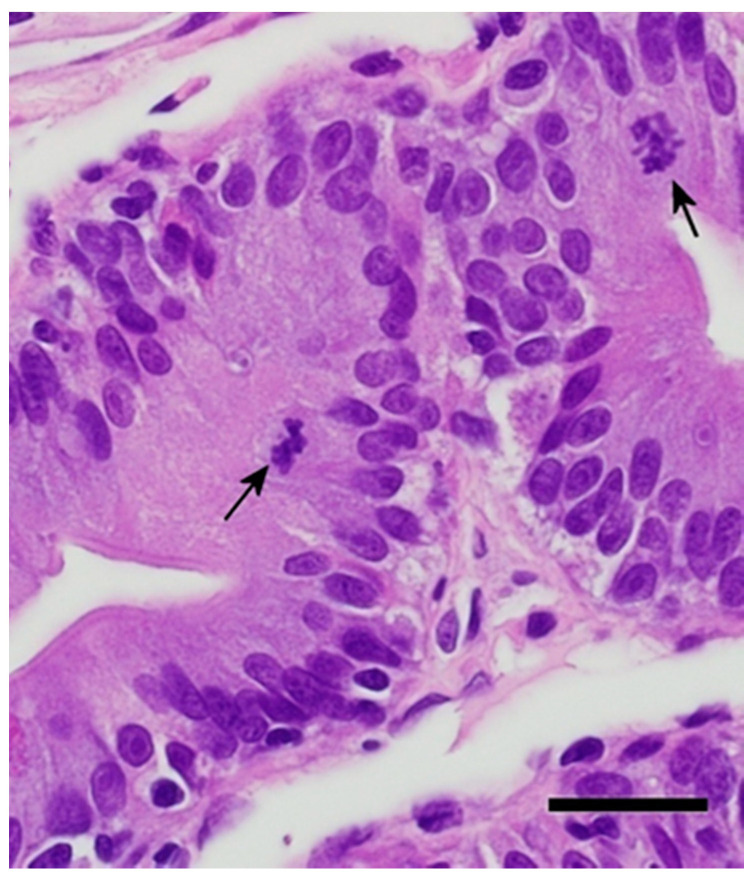
Four days post infection with early merogony (black arrows). Bar = 20 microns; magnification at 600×; hematoxylin and eosin.

Recognizable coccidian life stages were identified in the mucosal cells of the duodenum in 60% (3/5; 95% CI: 0.18–0.99) of the Group 1 (four days post infection) dragons. Merogony was the only life stage observed in the examined sections. Meronts were immature, rare, and scattered throughout the duodenum, with a visible crescent shaped to round lucent area surrounding some of the organisms (parasitophorous vacuole). The number of life stages averaged approximately four per linear centimeter of mucosa. The observed meronts contained basophilic structures consisting of multiple, marginated nuclei with the surrounding basophilic cytoplasm extending out in pseudopod-like protrusions that formed a stellate basophilic body within the cytoplasm of the host cell (Figure 3). Under a higher magnification (600×), the stellate basophilic body was found to be comprised of multiple immature merozoites. In some instances, the merozoites could be observed surrounding a mass of basophilic cytoplasm or residual body. There was a minimal to mild infiltration of the mucosa with lymphocytes and plasmacytes, with lower numbers of eosin-staining granulocytes (heterophils and eosinophils) in all sections of the duodenum. Coccidia were not observed in any other areas of the gut or in any of the examined organs.

**Figure 4:**
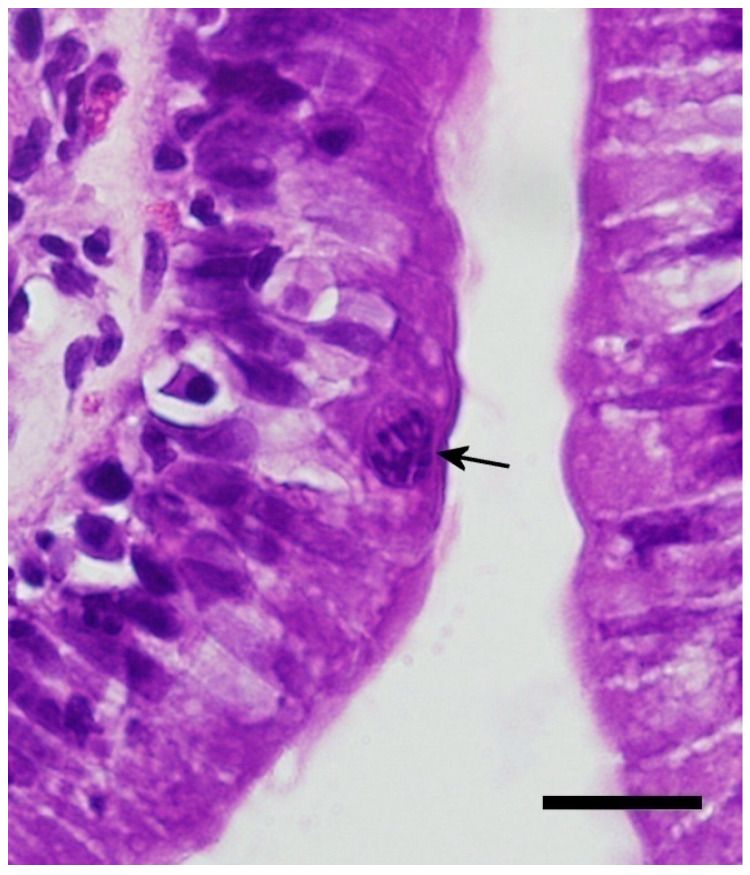
Eight days post infection with later stage first generation meront. Note the individual merozoites becoming more visible (black arrow). Bar = 20 microns; magnification at 600×; hematoxylin and eosin.

Coccidian life stages were also observed in 60% (3/5; 95% CI: 0.18–0.99) of the dragons from Group 2 (eight days post infection). The life stages consisted of immature and mature meronts (Figure 4) and a few immature microgamonts, which were characterized by clearly visible, multiple, marginated nuclei surrounding a deeply basophilic cytoplasm. Mild infiltrations of the mucosa with lymphocytes, plasmacytes, and scarce heterophils, and eosinophils were observed throughout the duodenum. There were 10–16 visible life stages per linear centimeter of mucosa. Some individuals had a mild vacuolation of infected and uninfected enterocytes. Vacuoles are non-staining and variable in their delineation, with some having distinct borders and others having more indistinct borders. No other life stages were observed in any of the other examined tissues or in extramucosal portions of the intestine. Life stages were only observed in the duodenum.

In Group 3 (12 days post infection), 80% (4/5; 95% CI: 0.45–0.99) of the animals had recognizable coccidian life stages. The life stages consisted of immature and mature meronts, as well as immature and mature or nearly mature macrogamonts and microgamonts. Zygotes (poorly walled immature oocysts) were also noted in some sections. There were 20–33 life stages per linear centimeter of mucosa. The mucosa had patchy areas that were moderately infiltrated with lymphocytes, plasmacytes, and lower numbers of heterophils and eosinophils. Enterocytes were frequently vacuolated in patchy areas with some variation in the degree of vacuolation among the individual animals. Enterocyte vacuolization was mild to moderate, with vacuoles having distinct margins. Life stages were only observed in the duodenal mucosa.

**Figure 5:**
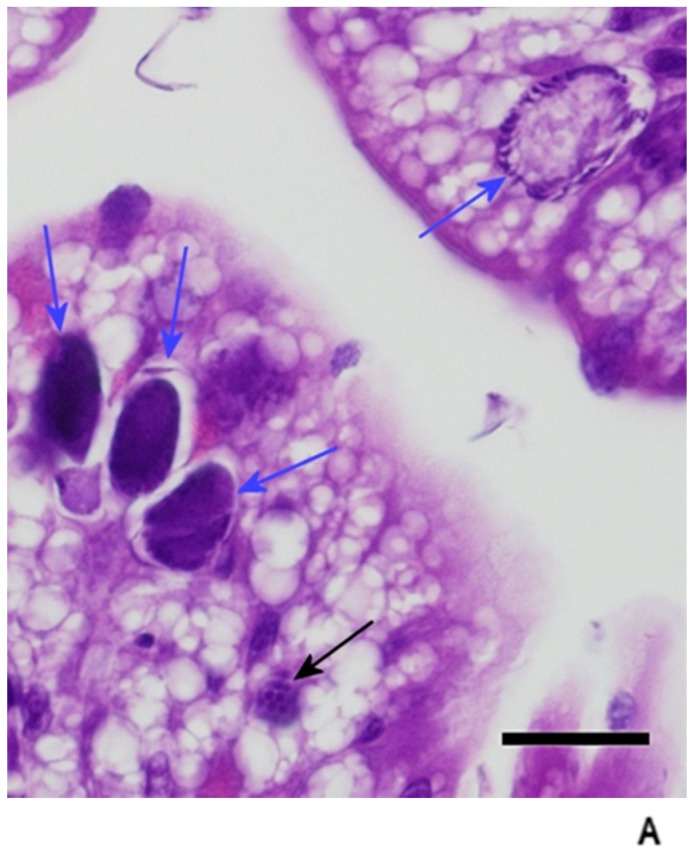

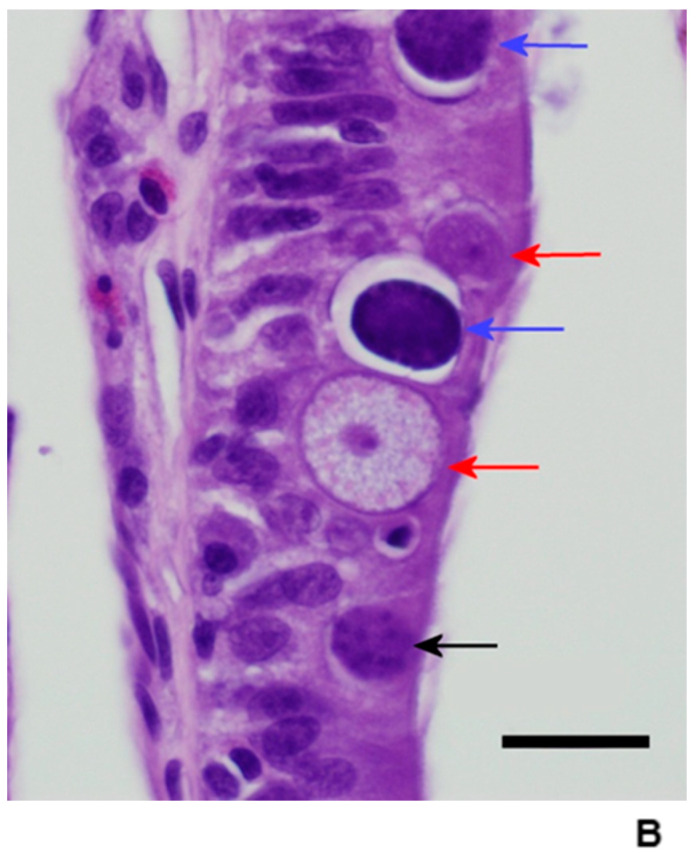
(**A**) Sixteen days post infection in the duodenum. Note the vacuolation of the enterocytes. (**B**) Sixteen days post infection in the distal duodenum. Note the low numbers of inflammatory cells (eosinophilic granulocytes) and good organization of the enterocytes still visible in this segment. Bar = 20 microns; magnification at 600×; hematoxylin and eosin. Black arrows = meronts; red arrows = macrogamonts; and blue arrows = microgamonts.

All five animals in Group 4 (16 days post infection) had recognizable coccidian life stages, including meronts, macrogamonts, microgamonts, and zygotes (Figure 5). One individual began shedding oocysts 15 days post infection. This individual also had a large number of zygotes and more mature unsporulated oocysts within its enterocytes. Enterocyte vacuolization was variable among the individuals, but it was generally more pronounced than in the previous groups. The degree of mucosal infiltration was variable but moderate overall, and there were patchy areas where infiltration by lymphocytes and plasmacytes was moderate to severe. There was a general increase in eosin-staining granulocytes (heterophils and eosinophils). Life stages were primarily observed in the duodenal mucosa, with lower numbers observed in the jejunum and ileum; rare life stages were observed in the colon, near the ileocolic junction, of three (60%) individuals. The number of life stages per linear centimeter was >500 in some sections. Some mucosal cells containing red-staining granules (zymogen type) were large, pronounced (hypertrophy), and more numerous, indicating hyperplasia.

**Figure 6:**
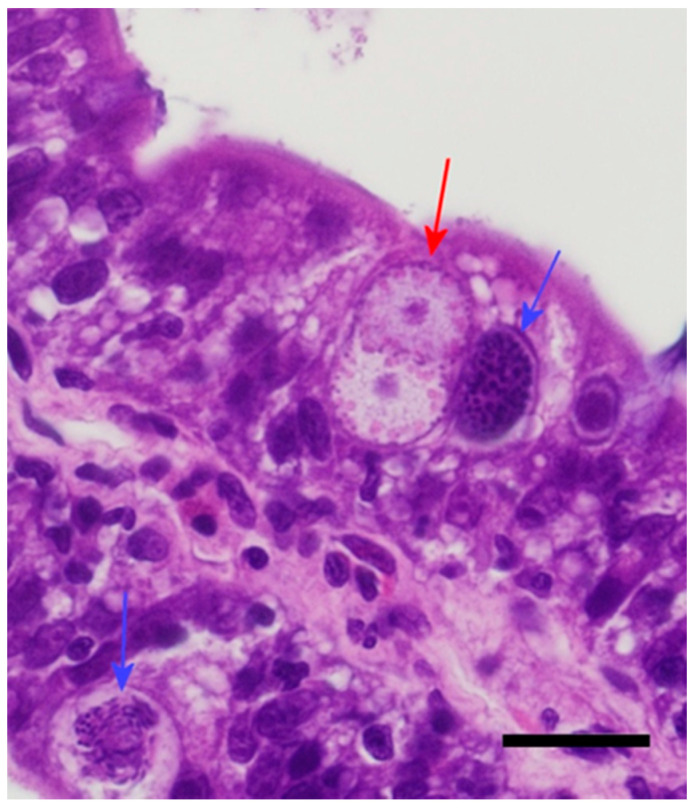
Twenty days post infection in the duodenum. Note the increased inflammation and the two macrogamonts with a young microgamont in “pseudosyzygy.” Bar = 20 microns; magnification at 600×; hematoxylin and eosin. Red arrows = macrogamonts; blue arrows = microgamonts.

**Figure 7:**
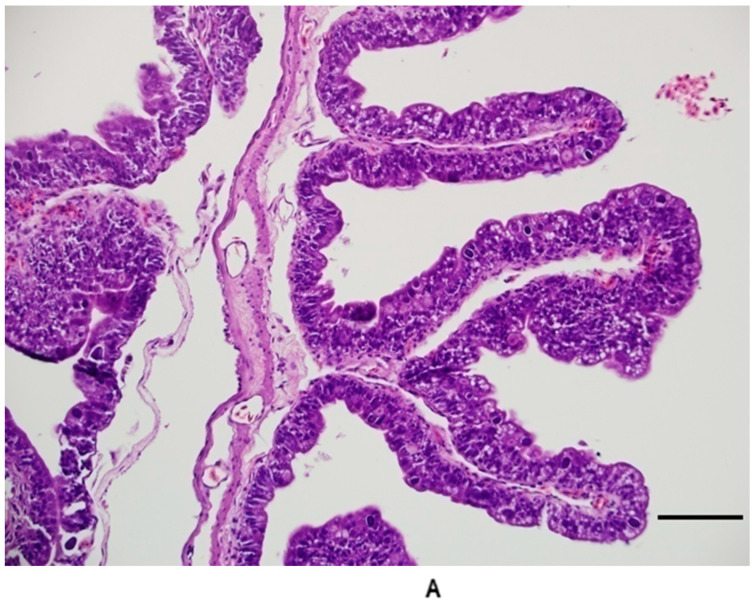

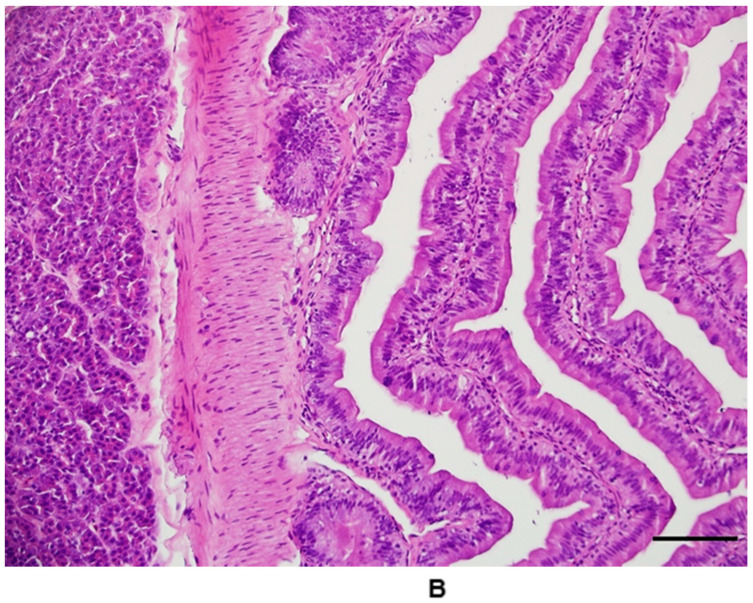
(**A**) Duodenum 20 days post infection. Note the short, attenuated villi and loss of the well-ordered appearance of the enterocytes. Branched villi and villi fused at the tips were common in late stages of infection in the duodenum. (**B**) Duodenum from control group animal for comparison. Bar = 100 microns; magnification at 200×; hematoxylin and eosin.

All five animals in Group 5 (20 days post infection) had recognizable coccidian life stages, and the histologic changes were similar to those observed in Group 4. The life stages were too numerous to count, and all stages were represented with a higher number of zygotes/oocysts than previous groups (Figure 6). Enterocyte vacuolization was severe in most individuals. The degree of leukocyte infiltration was moderate to severe; the majority were mononuclear cells (lymphocytes and plasmacytes), with lower numbers of eosin-staining granulocytes (heterophils and eosinophils). In the duodenum, the inflammation, in combination with the loss of enterocytes and the well-ordered arrangement of enterocytes, produced a disorganized appearance to the cells making up the villi (Figure 7). Numerous mucosal cells containing red granules (zymogen type) were pronounced in all individuals (hypertrophy and hyperplasia). Villi were noticeably shorter and blunted, and the fusion and branching of villi were frequently observed (Figure 7). All individuals had detectable oocysts in their feces by day 20 post infection. One individual began shedding 17 days post infection, two began shedding by 18 days post infection, one had detectable oocysts at 19 days post infection, and one had detectable oocysts 20 days post infection. Lower numbers of meronts, sexual stages, and oocysts were observed in the jejunum, ileum, and colon. The number of life stages per linear centimeter in the jejunum and duodenum were too numerous to count. The number of life stages per linear centimeter in the ileum ranged between 200 and 500. In the colon near the ileocolic junction, 20–80 life stages were observed per linear centimeter. Life stages were not observed in the middle or distal colon, nor were they found in extra-intestinal tissues.

Gross changes in the feces were not observed among the study groups. No animals developed diarrhea or had visible blood in the stools. Hemorrhagic areas (hemorrhagic enteritis) in the intestine were not observed in any of the individuals within the study.

There was no significant (*p* = 0.11) difference in tissue tropism when evaluating all sections of the intestinal tract for the presence of *I. amphiboluri*. There was no significant difference (*p* = 0.4) in the presence of life stages in the duodenum over time, as they were present at all time points; however, there were significant differences found for the jejunum, ileum, and colon (*p* = 0.0001, 0.001, and 0.006, respectively). Fisher’s exact tests found that life stages were significant less likely to be found in the 4, 8, and 12 day post infection groups compared to the 16 and 20 day post infection groups in the jejunum (*p* = 0.0079), the 20 day post infection group for the ileum (*p* = 0.008), and the 20 day post infection group for the colon (*p* = 0.047).

## 4. Discussion

### 4.1. Prepatent Period

*I. amphiboluri* exhibits a typical life cycle for enteric coccidia, with a slightly variable prepatent period. The results of this study also suggested that *I. amphiboluri* follows a homoxenous life cycle. Whether an intermediate host plays a role in this life cycle will require additional study, as this was not examined.

The prepatent period in this study ranged from 15 to 22 days, with most animals shedding detectable oocysts between 17 and 20 days post infection. Once shedding was confirmed, the infected dragons continued to shed oocysts in their feces over the remainder of the study. Unlike mammals that defecate daily, the majority of the bearded dragons defecated every three days. This variability could have accounted for the large range noted between dragons regarding the first appearance of oocysts in the dragon feces, as well as the difference noted in the early shedding between the two trials. The prepatent period could also have been affected by the number of infectious oocysts ingested or the number of oocysts that excysted. If low numbers of oocysts were ingested, the number of early oocysts would be expected, even with amplification through merogony, to be relatively low. Higher doses of oocysts may produce a much higher number of shed oocysts and lead to earlier detection, effectively pushing the prepatent period back by several days. This possibility is supported by the observation that higher doses of oocysts caused an increased oocyst output and more severe disease in calves [30]. However, Krassner [31] noted that exceptionally large doses of *Eimeria acervulina* oocysts in chickens led to a reduced number of oocysts. This finding was presumed to be due to a “crowding factor,” in which insufficient numbers of enterocytes were available for infective oocysts. This suggests that there is a threshold at which point oocyst numbers will stop increasing and begin to decrease with different levels of infection. The number of oocysts that excyst can also impact the prepatent period. In any group of infectious organisms, it is expected that some will be nonviable or fail to properly free themselves from the oocyst or sporocysts. This has been observed in poultry where oocysts failed to excyst and pass through into the feces [2]. Unfortunately, this “performance rate” for *I. amphiboluri* in bearded dragons is not known. Host immune function must also be considered, as some coccidia are killed by host defenses. If these different limitations are considered with the fact that the animals did not defecate daily, the variable prepatent period begins to be more understandable. However, the appearance of oocysts in the gut at the microscopic level can be used to shed some light on the extreme number of variables that seem to confound the question of prepatent period.

The best evidence for the prepatent period is the appearance of oocysts in the histologic sections. Some individuals in Group 3 (12 days post infection) demonstrated early stages of oocyst formation, while some of the animals in the fourth and fifth groups had feces collected after the 12th day, and the earliest detectable shedding occurred in one animal on day 15, matching the prepatent period study. This suggests that the actual shedding of oocysts begins after 12 days post-infection, but detectable levels are not reached until later. Therefore, a conservative estimate for most clinical practitioners would be to place the prepatent period at a minimum of 15 days. A conservative maximum must also take into account the infrequent defecation cycle. One animal did not shed detectable oocysts on day 20 but did have detectable oocysts on day 22. This then places the conservative maximum at 25 days post infection for the prepatent period.

Knowledge of the prepatent period is important when working with breeding operations, which often purchase stock from other herpetoculturists. In these cases, biosecurity is crucial if the resident population is free of *I. amphiboluri* and the new stock is of unknown status. An effective quarantine is necessary to prevent the introduction of coccidia into the resident population. This can only be accomplished by understanding the prepatent period. The effectiveness of a treatment, as well as the occurrence of re-infection, can also be better evaluated if the prepatent period is known. Animals where treatment resulted in negative floats immediately post treatment but that resumed shedding due to the resistance or early cessation of treatment cannot be distinguished from animals that have been successfully treated and re-infected from the environment unless the prepatent period is known.

### 4.2. Pathogenesis of Isospora Amphiboluri

In the first three groups, detectable life stages of coccidia could not be identified in all of the subject animals. This may have been due to several factors. All of the animals came from the same pool of animals and were approximately the same age; therefore, acquired age-related immunity is unlikely. All study animals had been previously naturally infected with *I. amphiboluri* and cleared with oral ponazuril, which suggests that there could have been some acquired immunity from previous challenge; however, the group used for the prepatent study was also previously naturally infected and treated, but all infected animals shed oocysts. Additionally, all study subjects in Groups 4 and 5 were infected and shed oocysts. The researchers’ clinical observations with infected colonies cast some doubt on acquired immunity, since no evidence of clearing or resistance has been observed in untreated animals in colonies where the parasite has become endemic. It is possible that the failure to infect some of the animals occurred with regards to the preparation and dosing (number of oocysts) of the inoculums; however, this was standardized across the entire study. Finally, it is possible that there was another disease process occurring at the level of the intestines or that the coccidia were outcompeted by other microorganisms in the intestine, although no specific pathology could be found during the necropsy of these animals to confirm any specific disorder.

The 12, 16, and 20 day post-infection groups showed an increasing prevalence over time, with all animals containing recognizable life stages in the 16 and 20 day post-infection groups. This finding may indicate that the initial number of infecting oocysts was too low or that too few oocysts excysted and infected some animals for life stages to be visible without amplification from rounds of merogony. In addition, despite using the same collection and handling techniques for the preparation of infectious doses, errors or problems with the doses themselves resulting in the death of oocysts cannot be completely ruled out.

The infection pattern identified in this study suggests that following excystation in the duodenum, the sporozoites invade the local mucosal cells and begin merogony (schizogony), with the subsequent release of merozoites and multiple rounds of merogony before entering gametogony. The parasite demonstrated no clear preference for an area of the intestine, but it seemed to progress down the intestine as the infection progressed. The exact number of asexual reproductions could not be determined, but given that merogony was observed from 4 to 20 days post infection, at least three, and possibly more, rounds are likely. Further investigation using a larger population of dragons with stages examined at 24 h intervals, rather than four day intervals, may be beneficial in elucidating the exact number of asexual amplifications. The presence of merogony at the later stages might also indicate repeat exposure. All animals shed low numbers of oocysts immediately after infection. The authors presume that this was due to oocysts failing to excyst and passing unchanged through the gut. Some of these oocysts would presumably then reside in the environment and possibly result in continued infection if the oocysts remained viable and had failed to excyst due to incomplete sporulation at the time of infection. Oocysts that have failed to excyst in poultry have been found to be viable, with some containing motile sporozoites [32].

The absence of life stages observed in the stomach, liver, lungs, kidney, spleen, heart, intestinal lamina propria, intestinal submucosa, intestinal muscularis, and skeletal muscle suggests that *I. amphiboluri* is a gut mucosa-limited species. All life stages were observed in the enterocyte cytoplasm.

The increased inflammation and hypertrophy observed in the secreting cells of the intestines were consistent with the expected immune response of the host as it attempts to eliminate the parasite. Changes observed in poultry have some similar characteristics, with moderate inflammation early in infection and a marked increase in inflammation as the infection progresses [2]. The vacuolar changes observed in the mucosa are of uncertain etiology. The mild vacuolation of the enterocyte cytoplasm was observed in patchy areas in all groups and may reflect either a normal enterocyte morphology or a fixation artifact. The more severe changes observed in late stage infection were limited to the duodenum and jejunum, with little or no vacuolar change in the distal intestinal enterocytes. This change may represent an osmotic change in the intestine resulting from the loss of enterocytes and the possible malabsorption or exudation of fluid into the damaged intestine with a resulting uptake by enterocytes.

## 5. Conclusions

In conclusion, the results of this study suggest that *I. amphiboluri* is a gut-limited coccidian with no intestinal site preference. The prepatent period is variable, but a conservative estimate places it in the range of 15–25 days. Animals that do not shed oocysts after 25 days may be considered negative; however, low-level infection with shedding of rare oocysts that could be missed on fecal floatation cannot be completely ruled out. Thus, a longer quarantine period with weekly repeated floats over the course of an additional two-to-three weeks may be advisable for those concerned about biosecurity. The organism is limited to the intestinal mucosa and capable of infecting enterocytes of the small and large intestine, with a progressive movement down the intestinal tract as the infection progresses. Pathologic changes are limited to the intestine in acute juvenile infections, though long-term infection over months and in adult animals should be examined. The further elucidation of the life cycle with 24 h sampling may be useful to determine the exact number of asexual amplifications.

## Data Availability

The data presented in this study are available on request from the corresponding author.
